# Inconsistent Condom Use among Iranian Male Drug Injectors

**DOI:** 10.3389/fpsyt.2013.00181

**Published:** 2014-04-04

**Authors:** Shervin Assari, Mosaieb Yarmohmmadi Vasel, Mahmood Tavakoli, Mahmoud Sehat, Firoozeh Jafari, Hooman Narenjiha, Hassan Rafiey, Khodabakhsh Ahmadi

**Affiliations:** ^1^Department of Health Behavior and Health Education, University of Michigan School of Public Health, Ann Arbor, MI, USA; ^2^Center for Research on Ethnicity, Culture, and Health, University of Michigan School of Public Health, Ann Arbor, MI, USA; ^3^Department of Psychology, Bu-Ali Sina University, Hamedan, Iran; ^4^Substance Abuse and Dependence Research Center, University of Social Welfare and Rehabilitation Sciences, Tehran, Iran; ^5^Universal Network for Health Information Dissemination and Exchange, Tehran, Iran; ^6^Medicine and Health Promotion Institute, Tehran, Iran; ^7^Center for Behavioral and Social Research, Darius Institute, Tehran, Iran; ^8^Behavioral Sciences Research Center, Baqiyatallah Medical Sciences University, Tehran, Iran

**Keywords:** inconsistent condom use, protective health behaviors, HIV risk behaviors, men, Iran, drug injectors

## Abstract

**Objectives**: The aim of this study was to determine the prevalence and associated factors of inconsistent condom use among Iranian male injecting drug users (IDUs).

**Materials and Methods**: Data came from the national Iranian behavioral survey of drug dependence, which sampled 7743 individuals with drug dependence, from medical centers, prisons, and streets in 29 provinces in Iran, in 2007. This study included all individuals who were male, IDUs, and were sexually active (*n* = 1131). The main outcome was inconsistent condom use which was assessed using a single item. A logistic regression was used to determine the association between socio-economic data, drug use data, and high risk injection behaviors with inconsistent condom use.

**Result**: 83.3% of sexually active IDUs (*n* = 965) reported inconsistent condom use. Based on the logistic regression, likelihood of inconsistent condom use was higher among those with a history of syringe sharing [Odds Ratio (OR); 1.63, 95% Confidence Interval (CI); 1.13–2.34], but lower among those with higher education levels (OR; 0.34, 95% CI; 0.14–0.82), those who mostly inject at home (OR; 0.09, 95% CI; 0.02–0.47), and those with a history of treatment (OR; 0.54, 95% CI; 0.31–0.94).

**Conclusion**: Because of the link between unsafe sex and risky injecting behaviors among Iranian IDUs, combined programs targeting both sexual and injection behavior may be more appropriate than programs that target sexual or injection behavior. The efficacy of combined programs should be, however, compared with traditional programs that only target sexual or injection behavior of IDUs.

## Introduction

With a population of 68 million, Iran has the highest per capita opiate consumption rate in the world (1). The Iranian Ministry of Health, Treatment and Medical Education has recently announced an estimated figure of 200,000 injecting drug users (IDUs) in Iran (2). It is believed that most Iranian IDUs are male, single and in the age range of 20–39 years old with more than 70% having a history of imprisonment and a history of addiction treatment. Most Iranian IDUs inject opioids, heroin, and crack (3).

Although Iran has a number of harm reduction programs for tackling HIV, HBV, and HCV epidemics among IDUs, prevalence of HCV and HIV infections in IDUs are still high. According to one study, 65.9 and 18.8% of Iranian IDUs are infected with HCV and HIV/AIDS infections, respectively (3). It is believed that IDUs account for 62% of new cases of HIV/AIDS in Iran (4). A qualitative study of HIV risk behaviors in Iran showed drug-related harm in almost all study locations. The study reported a prevalence of sharing injection instruments ranging from 30 to 100%. The study concluded that drug injection in Iran is strongly associated with HIV risk, and sharing injection instruments is a common behavior among Iranian IDUs (5). Other studies have also shown high rates of HIV risk behaviors among Iranian IDUs (6–12).

Designing condom promotion programs needs research based information on rate and determinants of inconsistent condom use among high risk populations (13). Such information is believed to have emerging public health implications (14) by increasing the efficacy of programs that target HIV transmission through sexual behaviors.

Policy makers and program planners have shown an increasing interest on epidemiology of condom use among the general population (15), people with sexually transmitted infections (16), HIV positive patients (17), sex workers (18), drug users (19), and IDUs (20). Most IDUs are known to be sexually active and engage in high risk sexual behaviors that facilitate HIV transmission from IDUs to the general population (21).

As most previous reports on risk profile of Iranian IDUs have mainly or exclusively focused on injecting behaviors (5–7, 10–12, 22), we aimed to investigate the prevalence and associated factors of inconsistent condom use among a nationwide sample of sexually active Iranian male IDUs.

## Materials and Methods

### Design and setting

Data came from a national behavioral survey of drug dependence, which sampled adults with drug dependence disorder (according to the Diagnostic and Statistical Manual of Mental Disorders – IV) from 29 provinces in 2007. This study was conducted under the financial aid of the Drugs Control Headquarters. Some other reports have been extracted from this database (6, 23–27).

The study was approved by the ethical review committee of the University of Social Welfare and Rehabilitation Sciences. Informed consent was obtained from all the participants after they had been verbally reassured that the information would be kept confidential.

### Sampling of main survey

Participants of the main survey were sampled from treatment centers (*n* = 1217), prisons (*n* = 584), and streets (*n* = 5860) of the capitals of 29 provinces in the Islamic Republic of Iran. In 82 (1%) individuals, setting was missing. The samples from treatment centers were selected with a simple random strategy from newcomers, using computer generated random numbers. Prisoners were also sampled randomly from those who were registered into the prison within the previous month. The snowball approach was used to take samples from streets. The number of samples taken from every province was proportional to the whole population of the province. This sampling strategy has been used as the main sampling strategy of drug dependence disorder surveys. The study used age, sex, marital status, and drug use data as a strategy to check if all individuals were different. Participants did not have identical data in these regards.

### Participants enrolled to this analysis

Although the mother study had sampled 7743 individuals, as most participants were not IDUs, only 1131 male current IDUs who were sexually active enrolled in this study. One hundred and sixty-seven (14.8) were selected from treatment centers, 846 (74.9%) were selected from streets, and 89 (7.9) were selected from prisons. In 2.4%, setting was missing. From 7743, 2091 (27%) were males who had positive history of intravenous injection. From this number, only 1131 (54%) participants’ current and main mode of drug use was injection. We only enrolled men in this analysis because <5% of IDUs were female.

### Process

Data collection started in April 2007 and lasted for 5 months. The interviews were carried out by university graduates with drug use research interest who were dispatched to the provinces after being trained through workshops in Tehran (the capital of Iran). Each interview took between 60 and 90 min. Data were collected using a paper-based questionnaire that was the modified version of the one used in the previous national drug dependence survey of Iran. Sixty nine items were classified in nine different parts including: (1) socio-economic data (at the time of data collection), (2) family data, (3) first use data, (4) lifetime drug use, (5) current drug of dependency, (6) injection data, (7) high risk behavior, (8) treatment data, and (9) social network. The questionnaire is available online (23).

Independent variables in the current study included socio-economic data (at the time of data collection), family data, first use data, injection data, high risk behavior data, and treatment data. Some of the variables included age, gender, educational level, marital status, living condition, homelessness, employment status, income, sources of income, family history of substance use, first place of drug use, first situation of drug use, person who suggested drug use, common place of injection, history of treatment, syringe sharing, history of arrest, and imprisonment.

To make the final costs internationally comparable, drug costs were presented in purchase power parity or international Dollar (PPP$). The conversion rate for PPP$ was equal to 2727 Rials according to the World Bank database (28).

### Outcome

The main outcome was inconsistent condom use, asked by the following question: how often do you use condoms? Answers included: (1) often, (2) half the times, (3) less than half of the times, and (4) seldom, and (5) never. A single item five-point scale has been previously used for condom use (29, 30). We considered inconsistent condom use as any answer except often. Although some researchers believe that self-reported condom use may have limited accuracy (31), high reproducibility and validity has also been reported (32, 33).

### Statistical analysis

The data were analyzed using Statistical Package for the Social Sciences (SPSS) for Windows 13. First, we checked the normality of numerical data; with the use of a One-Sample Kolmogorov–Smirnov test. In order to present numerical data with a normal distribution, we used mean and standard deviation (SD) and for data with skewed distribution, we presented the median, and the first and third quartiles. In order to compare categorical variables between those with and without “inconsistent condom use,” a Chi-square test was used. The comparison of age between the two groups was done with a *t*-test, and the comparison of monthly family income and expenditures of drug use between two groups was done with a Mann–Whitney. Multivariate stepwise logistic regression was applied to determine the associated factors of inconsistent condom use. We used backwards elimination for our regression analysis. All variables with a significant association with inconsistent condom use in bivariate analysis were put into the stepwise model. The Odds Ratio (OR) and 95% Confidence Interval (CI) were reported. *p*-value <0.05 was considered significant.

## Results

All participants were male, with mean (SD) ages at study, at first drug use and at first injection being 31.3 ± 8.3, 18.6 ± 5.4, and 25.9 ± 6.7 years, respectively. Most participants were Muslim, lived in urban areas, and had not completed high school (Data not shown).

### Outcome

Out of 1131 sexually active IDUs, 965 (83.3%) reported inconsistent condom use.

### Associates of inconsistent condom use

Inconsistent condom use was not associated with age at study, age at first drug use, or age at first injection. IDUs with inconsistent condom use had a lower monthly income (median: 200, first quartile: 110, third quartile: 350 vs. median: 250, first quartile: 150, third quartile: 400, *p* = 0.001) and lower median monthly money paid for drugs (median: 150, first quartile: 100, third quartile: 270 vs. median: 200, first quartile: 120, third quartile: 350, *p* < 0.001). The association between inconsistent condom use and socio-economic variables are presented in Table 1.

**Table 1:** **Associations between socio-economic data and inconsistent condom use**.

		Inconsistent condom use		p*
		*n*	%	
Education level	Illiterate or were barely able to read and write	105	90.5	0.001
	Primary school to diploma	783	83.6	
	Upper diploma	54	70.1	
Marital status	Single	496	82.4	0.047
	Married	293	81.8	
	Separate, divorce, and widow	170	89.5	
Being homeless	No	790	82.3	0.016
	Yes	137	90.1	
Living alone	No	778	82.3	0.053
	Yes	187	87.8	
Jobless	No	548	80.8	0.007
	Yes	417	86.9	
Drug income	No	654	81.2	0.007
	Yes	294	87.8	
Job income	No	462	85.6	0.040
	Yes	486	81.0	
Illegal income	No	604	80.9	0.004
	Yes	344	87.5	

*Chi-square.

Inconsistent condom use was higher among IDUs whose dominant drug was heroin in comparison to IDUs that had a different dominant drug (86.3 vs. 81.3%, *p* = 0.025). Inconsistent condom use was not associated with poly drug use. Inconsistent condom use was lower in IDUs with history of drug use treatment (82.2 vs. 89.2%, *p* = 0.010). The association between inconsistent condom use and drug-related variables are presented in Table 2.

**Table 2:** **Bivariate associations between drug-related variables and inconsistent condom use**.

Usual place of injection		Inconsistent condom use		p*
		*n*	%	
Own home	No	478	86.9	0.002
	Yes	487	80.1	
Ruin	No	659	81.9	0.043
	Yes	306	86.7	
Student house	No	963	83.7	<0.001
	Yes	2	25.0	
Work place	No	863	84.3	0.017
	Yes	102	76.1	
Prison	No	904	82.7	0.019
	Yes	61	93.8	

*Chi-square.

Among IDUs, inconsistent condom use was associated with having sex with multiple partners (19.1 vs. 11.7%, *p* = 0.001). Inconsistent condom use was higher in IDUs who reported syringe sharing (87.2 vs. 79.5%, *p* = 0.001). Inconsistent condom use was not associated with history of lifetime non-fatal overdose, non-fatal overdose in the past year, arrest history in the past year, or imprisonment in the past year (*p* > 0.05).

Religion (p=0.0652), Living place (p=0.669)

Having legal income coming from a source other than a job (*p* = 0.382), income through selling furniture (*p* = 0.766), lifetime history of tobacco smoking (*p* = 0.216), cigarette smoking by parents (*p* = 0.937), cigarette smoking by other members of the family (*p* = 0.728), drug use by parents (*p* = 0.139), and drug use by members of the family (*p* = 0.491) were not associated with inconsistent condom use among IDUs. Most important reason for beginning drug use (*p* = 0.232) was not associated with inconsistent condom use among IDUs. First person who suggested substance (*p* = 0.501), first situation of drug use (*p* = 0.337), first place of drug use (*p* = 0.467), first place of injection (*p* = 0.722), first situation of first injection (*p* = 0.325) were not associated with inconsistent condom use among IDUs. Cause of first injection (*p* = 0.75), the person with whom the IDU injects (*p* = 0.143), and frequency of injection (*p* = 0.70) were not associated with inconsistent condom use among IDUs. Common place of injection as friends homes (*p* = 0.711), parks (*p* = 0.675), schools (*p* = 0.235), street and lane (*p* = 0.439), and soldiers’ camps (*p* = 0.526) were not associated with inconsistent condom use among IDUs.

### Logistic regression

Based on multivariate analysis, a higher likelihood of inconsistent condom use was associated with history of syringe sharing; however, a lower likelihood of inconsistent condom use was associated with high education level, home as usual place of injection and history of treatment of drug disorder (Table 3).

**Table 3:** **Associated factors of inconsistent condom use based on a logistic regression**.

	*p*	Odds ratio	95% Confidence interval for odds ratio	
			Lower	Upper
Education level (completed high school)	0.016	0.34	0.14	0.82
Common place of injection (home)	0.004	0.09	0.02	0.47
Syringe sharing	0.008	1.63	1.13	2.34
History of treatment	0.029	0.54	0.31	0.94

## Discussion

Inconsistent condom use was reported by about 8 of 10 sexually active male IDUs in Iran. Inconsistent condom use was higher among IDUs who shared syringes, and among those with higher educational level, history of drug dependence treatment, and those who used to inject drugs inside their homes.

Literature on low rate of condom use among people who use drugs supports our finding (34). Inconsistent condom use among IDUs varies widely from <50% in Thailand (35) to more than 70% in Vietnam (36) and the United States (37). One study reported that 88% of IDUs had rarely or never used condoms during intercourse with sex workers, and that <10% had consistently used condoms during sexual relations with casual partners (38). Based on another study in Iran, 19% of female sex workers reported at least one occasion of unprotected sex with IDU(s) in the month preceding the study (7).

Low rates of consistent condom use by IDUs may be in part due to poor knowledge, or a consequence of sex under the influence of drugs. Many drug users take drugs prior to intercourse which may disturb their decision and condom use negotiation (39).

Our study showed that inconsistent use of condoms was higher among IDUs with lower levels of education and those who usually inject outside their homes. A link between low educational level and inconsistent condom use has been shown in many (40–42), but not all, studies (43). This link may be mediated by condom use knowledge or condom use negotiation skills.

We found a link between inconsistent condom use and syringe sharing. Similar reports have been reported from other geographic regions (44–46). This co-occurrence may be explained by similar risk factors and common pathways for different risky behaviors. It is also plausible to assume that one risky behavior may facilitate another risk taking behavior.

Our study also showed that inconsistent use of condoms was higher among IDUs who usually inject outside their homes. In fact, our study showed that injecting in one’s own home had the strongest relationship to consistent condom use of all of the variables examined. For another risky behavior, which is needle and syringe sharing among IDUs, our previous study in Iran has shown that being alone may have some protective roles (6). In general, characteristics such as location of drug use may be linked to a wide range of risky behaviors. In the study by Narenjiha et al., shared injection was linked to being alone at most injections (6).

It has been shown that a majority of IDUs may inject drugs in their own residence (47). Injecting alone in Iran has been reported to be associated with lower likelihood of needle sharing (48). In one study, 15% of IDUs reported always injecting alone. The study showed that IDUs who reported injecting alone were substantially less likely to report injection with a syringe or other drug preparation equipment previously used by another injector. Authors suggested that inviting injectors to inject alone may facilitate safe injection by granting the individual greater control over the injection setting (49).

Our study showed reduced inconsistent condom use among those IDUs who had attended drug treatment centers. Literature has both in line (50) and contradictory results (51, 52) that warrant further research. The link can be explained by the role of risk reduction interventions that are in place for IDUs as a part of treatment. Although we did not collect data on content of treatment programs that our participants had entered, our finding may reflect the effect of HIV testing or VCT programs that have a risk reduction component.

Most IDUs are sexually active (21), and their sexual behaviors place the community at risk for HIV/AIDS. Condom promotion programs that target IDUs should be considered as a part of HIV-AIDS prevention programs. Messages directed at IDUs should encourage them to consistently use condoms. Although harm reduction programs in Iran have reduced the rate of syringe sharing (45), high risk sexual behavior of IDUs is still a threat to the health of the nation.

Condom use offers protection against HIV/AIDS and other sexually transmitted infections (53). It is only the consistent use of condoms that effectively protects against HIV infection (54). As a result, surveys should continuously monitor the rate of inconsistent condom use among high risk populations, including IDUs (55).

Promotion of condom use can be achieved via different programs such as condom education and programs that increase access to condoms (54). Programs with a condom promotion component have proved to be effective in reducing high risk sexual behaviors (56).

There are some limitations to this study. First, the results rely on participants’ self-reported data which may be accompanied by information bias, not only in the form of recall bias, but also social desirability bias (57). Second, because of the cross-sectional design of this study, it is not possible to draw a conclusion on the direction of the associations. Third, this study did not ask about condom use with different types of sexual partners. Fourth, the study did not assess knowledge and attitude about condom use, reasons for not using condoms, perceived need for condom use, and availability of condoms (58). Fifth, the current study’s response options to the outcome did not include “always or almost always.” As a result, individuals who have reported “often” should not be considered as “always or almost always” using condoms. This may have also influenced the distribution of all responses to this outcome. This should be considered before any comparison of the rates with other studies, as operationalization of inconsistent condom use is very different in various studies. Sixth, the main study has not provided information on the total number of persons that were approached for an interview and how many refused. Finally, as we tested a large number of independent variables, our study should be considered as an exploratory study. The result is, however, relevant to the epidemic of HIV in Iran and probably the Middle East, where such data are scant and poor (59).

## Conclusion

More than 8 of 10 Iranian sexually active male IDUs report inconsistent condom use, which reflects a serious public health threat. Condom promotion programs in Iran should focus on IDUs who have a lower education level, who share syringes, who inject drugs outside their home and those with no previous treatment history. Further research should test the efficacy of combined programs that target both sexual and injection behavior of IDUs.

## Conflict of Interest Statement

The authors declare that the research was conducted in the absence of any commercial or financial relationships that could be construed as a potential conflict of interest.
